# The Study of a Novel Paeoniflorin-Converting Enzyme from *Cunninghamella blakesleeana*

**DOI:** 10.3390/molecules28031289

**Published:** 2023-01-29

**Authors:** Yiheng Ye, Hairun Pei, Xueli Cao, Xueying Liu, Zhanghan Li, Biying Wang, Yan Pan, Jimin Zheng

**Affiliations:** 1 Beijing Advanced Innovation Center for Food Nutrition and Human Health, Beijing Technology & Business University, Beijing 100048, China; 2 College of Chemistry, Beijing Normal University, Beijing 100875, China; 3 Beijing Vocational College of Agriculture, Beijing 102442, China

**Keywords:** biotransformation, *Cunninghamella blakesleeana*, paeoniflorin

## Abstract

Paeoniflorin is a glycoside compound found in *Paeonia lactiflora* Pall that is used in traditional herbal medicine and shows various protective effects on the cardio-cerebral vascular system. It has been reported that the pharmacological effects of paeoniflorin might be generated by its metabolites. However, the bioavailability of paeoniflorin by oral administration is low, which greatly limits its clinical application. In this paper, a paeoniflorin-converting enzyme gene (G6046, GenBank accession numbers: OP856858) from *Cunninghamella blakesleeana* (AS 3.970) was identified by comparative analysis between MS analysis and transcriptomics. The expression, purification, enzyme activity, and structure of the conversion products produced by this paeoniflorin-converting enzyme were studied. The optimal conditions for the enzymatic activity were found to be pH 9, 45 °C, resulting in a specific enzyme activity of 14.56 U/mg. The products were separated and purified by high-performance counter-current chromatography (HPCCC). Two main components were isolated and identified, 2-amino-2-p-hydroxymethyl-methyl alcohol-benzoate (tirs-benzoate) and 1-benzoyloxy-2,3-propanediol (1-benzoyloxypropane-2,3-diol), via UPLC-Q-TOF-MS and NMR. Additionally, paeoniflorin demonstrated the ability to metabolize into benzoic acid via G6046 enzyme, which might exert antidepressant effects through the blood–brain barrier into the brain.

## 1. Introduction

*Paeonia lactiflora* Pall is widely used in Chinese herbal medicine, with a long history of good safety [1]. Herbal formulations containing it, such as the patented Chinese drugs, “Xiaoyao-wan” and “Xiaoyao-san,” are prescribed for the treatment of depression-like disorders [2,3]. The pharmacological activities of the dried roots of *Paeonia lactiflora* Pall include alleviating pain, tonifying blood, and regulating menstruation [4]. Paeoniflorin is an important component of *Paeonia lactiflora* Pall that shows various protective effects on the cardio-cerebral vascular system, particularly in diabetes mellitus (DM)-associated macrovascular complications [5]. In addition, from a mouse study, it was postulated that the mechanism of the neuroprotective and antidepressant effects of paeoniflorin is to activate neuronal FGF-2/FGFR1 signal by inhibiting the hippocampal microglia [6]. Paeoniflorin is potentially an important drug candidate for antiapoptosis [7], anti-inflammation [8], the treatment of Parkinson’s and Alzheimer’s disease [9,10,11,12], and antioxidantation [13] and antidepressant activities [6,14,15].

Low bioavailability is a common limitation for many natural compounds with useful pharmacological effects [16]. Paeoniflorin, which can be mainly extracted from *Paeonia lactiflora* Pall, has a poor absorption when administrated orally. The kinetic parameters of paeoniflorin in plasma proved that it could rarely be transformed and absorbed in the stomach, liver, and intestinal wall [17,18,19]. Previous studies also show that paeoniflorin is mainly metabolized by gut flora, and the pharmacological effect of paeoniflorin may be produced by the resultant metabolites [20]. Therefore, the clinical applicability of paeoniflorin is greatly limited due to its low bioavailability and unrevealed pharmacological mechanism [19,21].

A study displayed that the degradation by gut microbiota enzymes may be one of the main reasons for the low bioavailability of paeoniflorin [14]. Another study of the absorption and transformation of paeoniflorin and other active components in Guizhi Decoction, a traditional Chinese herbal formula containing paeniflorin, in rat gastrointestinal flora showed that paeoniflorin was completely transformed in rat cecum and colon within 24 h [18]. This confirmed that the intestinal flora metabolizes paeoniflorin. It is therefore possible that paeoniflorin has a regulatory effect on gut microbiota that helps to alleviate depression [14]. The study of paeoniflorin metabolites and their intestinal bacterial metabolism is therefore of great significance for an in-depth understanding of its pharmacodynamic mechanisms.

Some microbial enzymes were found to possess relative substrate specificities. They usually catalyze reactions with a chemical bond type or stereo specificity. Due to the similarity between microbial metabolites and mammalian metabolites, microbial transformation has been used as an effective auxillary method to study the metabolism of some drugs or natural products in mammals [22,23,24]. Some studies have shown that some microorganisms can convert paeoniflorin, such as *Cunninghamella blakesleeana* (AS 3.970) [25], but no detailed mechanism has been reported so far. 

To further study the mechanism underlying the conversion activity of *Cunninghamella blakesleeana* (AS 3.970), here, we describe a key synthetase found in *Cunninghamella blakesleeana,* which can convert paeoniflorin into its metabolites. In this study, the *Cunninghamella blakesleeana* (AS 3.970) synthetase genem, G6046, was identified and sequenced using transcriptomics. In order to verify whether G6046 possesses the converting activity of paeoniflorin, we cloned, expressed, and roughly purified G6046 protein, and the enzymatic activity was evaluated in vitro. We confirmed that G6046 protein can indeed convert paeoniflorin into several metabolites, and the further purification of G6046 greatly improved the efficiency of the conversion of paeoniflorin. The converted products were separated and purified by high performance counter-current chromatography (HPCCC), and their structures were identified by UPLC-Q-TOF-MS and NMR analysis. In this study, we identified a novel paeoniflorin-converting enzyme and its products from *Cunninghamella blakesleeana*, which may provide a clue to study the microbial metabolism of paeoniflorin. 

## 2. Results

### 2.1. MS Analysis to Determine Exoenzyme for Paeoniflorin Transformation

**Figure 1:**
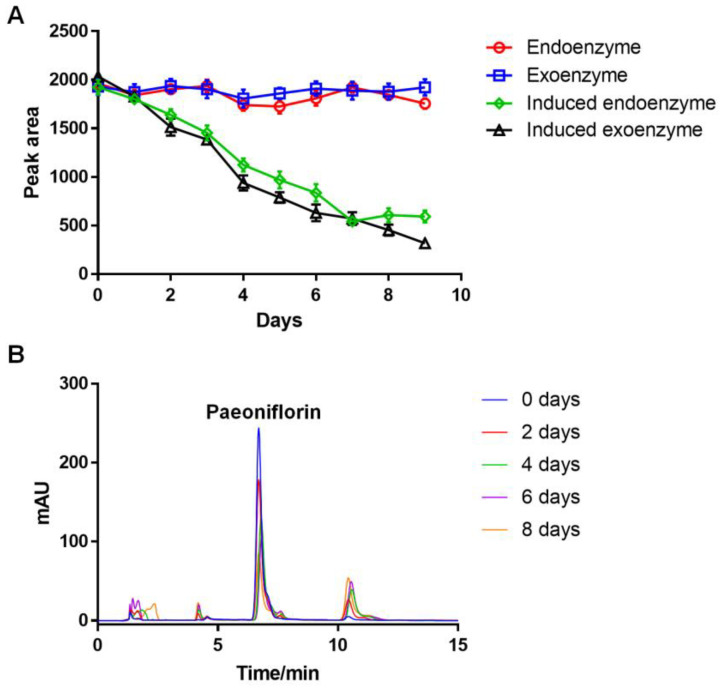
The conversion effects of the key enzymes. (**A**) 100 mL of the *Cunninghamella blakesleeana* (AS 3.970) medium was poured off and induced by adding 1.5 mL of paeoniflorin solution (1 mg/mL). The peak area of paeoniflorin was measured via HPLC. The endoenzyme was obtained from the supernatant after cell fragmentation and subsequent centrifugation, and the exoenzyme was the supernatant medium. Both the induced endoenzyme (green diamond) and exoenzyme (black triangle) by paeoniflorin had comparable conversion effects. (**B**) HPLC analysis of the conversion activity of the induced exoenzyme, obtained on an Agilent ZORBAX SB-C18 (4.6 × 250 mm, 5 μm); column temperature, 30 °C; mobile phase, H_2_O (0.2% phosphoric acid):methanol = 70:30 (*v*/*v*); flow rate, 1.0 mL/min; injection volume, 10 μL; detection wavelength, 230 nm. After 8 days of reaction, the paeoniflorin was converted by the induced exoenzyme.

*Cunninghamella blakesleeana* (AS 3.970) has been reported to convert paeoniflorin [25]. The endoenzymes and exoenzymes from paeoniflorin-induced *Cunninghamella blakesleeana* (AS 3.970) were separated and compared against the uninduced cultures to investigate the effects on paeoniflorin. The extent of paeoniflorin-converting activity of the endogenous and exogenous fractions was measured via high performance liquid chromatography (HPLC) and compared to determine which enzymes have more paeoniflorin-converting activity. The results showed that both the induced endoenzyme and exoenzyme by paeoniflorin had comparable conversion effects (Figure 1A). Therefore, the enzyme or enzymes that convert paeoniflorin were determined to be inducible enzymes that distribute both intracellularly and extracellularly.

**Figure 2:**
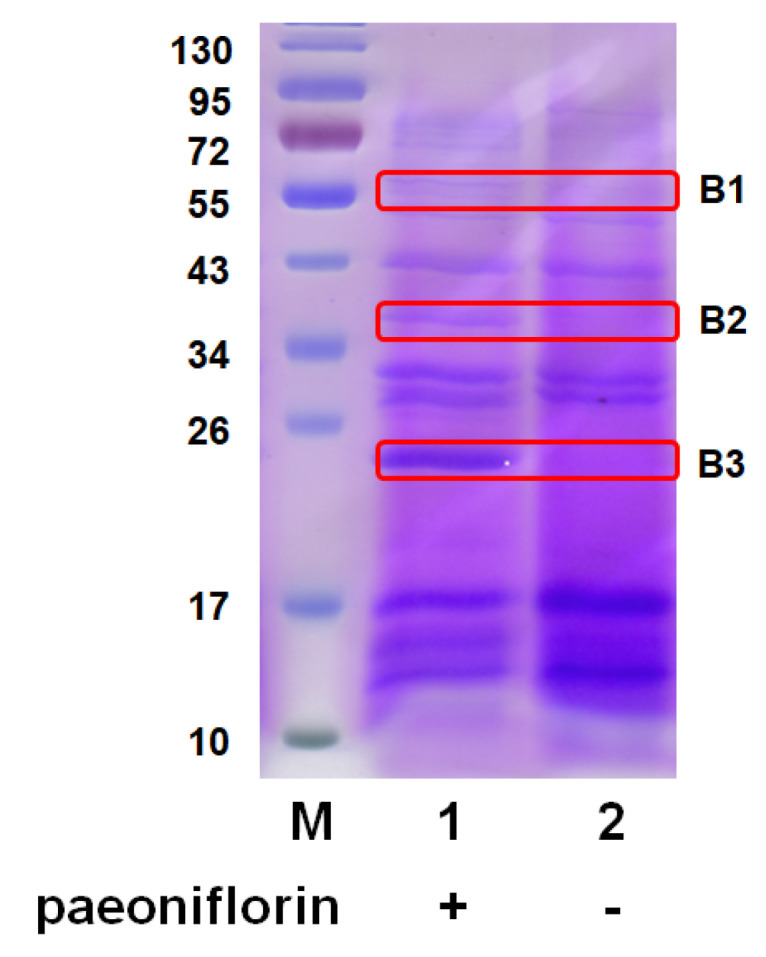
SDS-PAGE comparison of *Cunninghamella blakesleeana* (AS 3.970) exoenzyme with and without paeoniflorin induction. M: marker. Lane 1: induced exoenzyme with paeoniflorin. Lane 2: uninduced exoenzyme without paeoniflorin. B1, B2, and B3 were analyzed by MS.

To identify the key enzymes involved in the biotransformation of paeoniflorin, the proteins in AS 3.970 and culture medium with or without paeoniflorin induction were analyzed by SDS-polyacrylamide gel electrophoresis (SDS-PAGE). No difference could be observed in the bands of the gels between the two conditions due to the density of intracellular protein. Intriguingly, the extracellular proteins were relatively simple (Figure 2), and it was easy to observe the difference. Three distinctive bands were observed between the uninduced exoenzyme and the induced exoenzyme, designated B1, B2, and B3. The three bands were then cut and analyzed using MS (Tables S1–S3 Supplementary Material). MS analysis of B1, B2, and B3 indicated several candidates, including a phosphoesterase family-domain-containing protein, hypothetical protein, and rhizopuspepsin 4 precursor.

### 2.2. Transcriptomics Analysis of the Paeoniflorin-Induced Cunninghamella blakesleeana 

A transcriptomics analysis was performed on *Cunninghamella blakesleeana* (AS3.970) to analyze the induction effect of paeoniflorin. The results of AS3.970 before and after induction are shown in Table S4. Three samples were left uninduced U1, U2, and U3, and three, I1, I2, and I3, were induced with paeoniflorin. CleanReads was the total reads number of CleanReads after data quality control (one read represented two sequences, PE). The total number of bases of CleanReads after data quality control was represented as CleanBase. CleanQ20 (%) represented the proportion of bases with a base quality value greater than 20 (error rate less than 1%) in the total number of bases in CleanReads after data quality control. The ratio of bases with a base quality value greater than 30 (error rate less than 0.1%) to the total bases in CleanReads after data quality control was expressed as CleanQ30 (%). CleanGC (%) represented the ratio of bases G and C to total bases in CleanReads after data quality control. Particularly, all the values of CleanQ20 (%) were higher than 96 and all the values of CleanQ30 (%) were over 91. The small error rate indicated that the results were reliable.

Compared to uninduced AS3.970, a total of 3051 genes were changed at the transcription level in paeoniflorin-induced AS3.970, of which 1975 genes were up-regulated and 1076 genes were down-regulated (Table S5). As the key enzyme was produced after paeoniflorin induction (Figure 1A), the following research was focused on the 1975 up-regulated genes. 

### 2.3. Comparative Analysis between Protein MS and Transcriptomics

**Figure 3:**
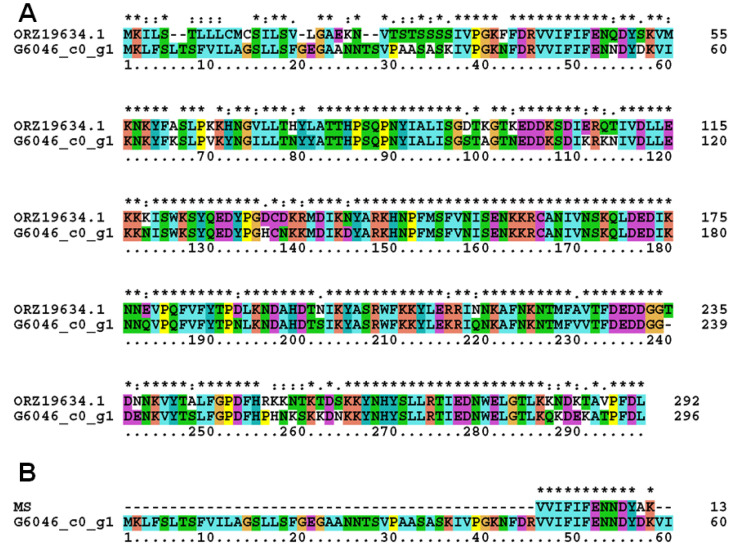
Amino acid sequence alignment. (**A**) Comparison of the amino acid sequence of ORZ19634.1 and G6046_c0_g1. (**B**) One amino acid sequence, VVIFIFENNDYAK, of MS results was matched with the amino acid sequence encoded by the G6046_c0_g1 gene. * indicates positions which have a single, fully conserved residue, : indicates that one of the following “strong” groups is fully conserved.

Comparing the MS results of the paeoniflorin-induced bands and transcriptomics results of the up-regulated genes after paeoniflorin induction found the NR_ID in common to be ORZ19634.1 (phosphoesterase family-domain-containing protein (*Absidia repens*), Table S2). Transcriptomics results encode NR_ID as ORZ19634.1 and its GeneID was G6046_c0_g1. In order to verify the accuracy of the analysis results, the G6046_c0_g1-encoded amino acid sequence was compared with the amino acid sequence of ORZ19634.1 (Figure 3A). One amino acid sequence, VVIFIFENNDYAK, was identified in the mass spectrum result of ORZ19634.1. The sequence was matched with the amino acid sequence encoded by the G6046_c0_g1 gene (Figure 3B). Therefore, the gene with GeneID of G6046_c0_g1 may code the key enzyme for paeoniflorin transformation in AS3.970, and we designated this enzyme G6046. The calculated molecular weight and theoretical pI of G6046 were 34 KD and 9.17, respectively. 

### 2.4. Cloning of Full-Length G6046 and Its Truncations

**Figure 4:**
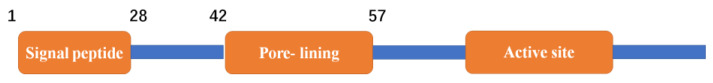
A schematic drawing of the functional domain of G6046. G6046 possesses a signal peptide and a pore-lining helix domain at its N-terminus.

As shown in Figure 4, secondary structure prediction of G6046 revealed a signal peptide and a pore-lining helix domain at the N-terminus. Based on this, recombinant vectors for full-length and truncated G6046 were designed. Among them, G6046 truncations were designed by deleting the 57 or 82 N-terminal residues. To achieve better gene expression in *E. coli*, full-length and truncated constructs were fused with N-terminal MBP or T4L tags, producing the following constructs: thus constructed the MBP-G6046, T4L-G6046, MBP-G6046-NΔ57, T4L-G6046-NΔ57, MBP-G6046-NΔ82, and T4L-G6046-NΔ82 clones. pET22b and pET28b were selected as the gene vectors to achieve better expression of G6046 and its truncations. The details of recombinant G6046 are listed in Table 1. 

### 2.5. Expression and Purification of G6046 from E. coli

**Figure 5:**
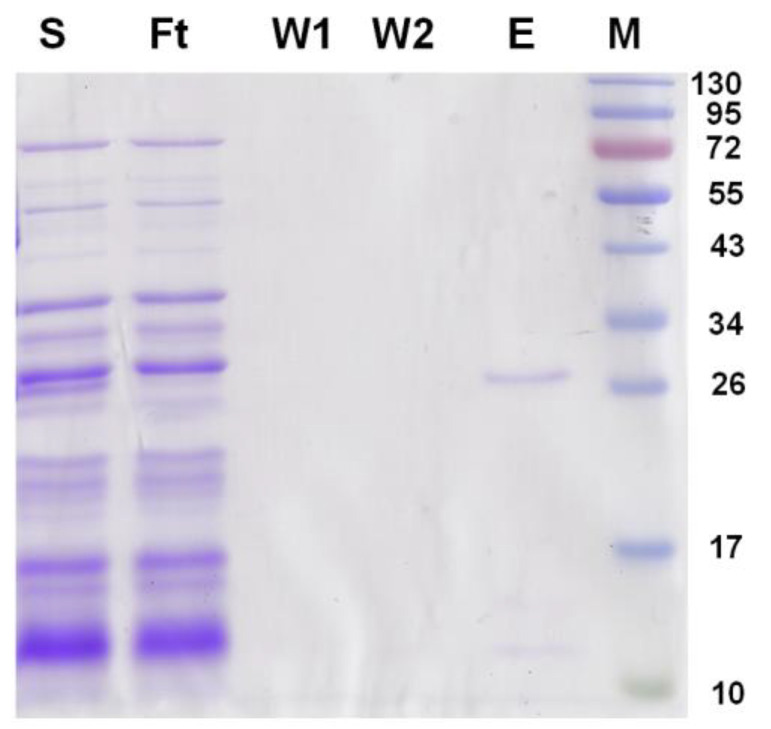
SDS-PAGE analysis of expression and purification of G6046-NΔ82-22b. Lanes S and Ft: the supernatant and flowthrough of G6046-NΔ82-22b; lanes W1 and W2: samples from the buffer wash; lane E: the eluted samples from the resin; lane M: molecular weight marker.

Vectors containing full-length and truncated G6046 genes were expressed in *E. coli* BL21 (DE3) strains. However, full-length G6046 was expressed as an inclusion body in *E. coli*, both when using pET22b and pET28b vectors. The T4L- and MBP-tagged constructs also expressed as inclusion bodies. This may have occurred because the signal peptide and the pore-lining helix domain in G6046 did fold correctly in *E. coli*, however, G6046-NΔ57, which lacked these domains, still expressed as an inclusion body whether recombined into different vectors or fused with MBP or T4L tags. The only construct that successfully expressed a G6046 truncation was G6046-NΔ82 in pET22b (Figure 5). 

### 2.6. Activity Assay

**Figure 6:**
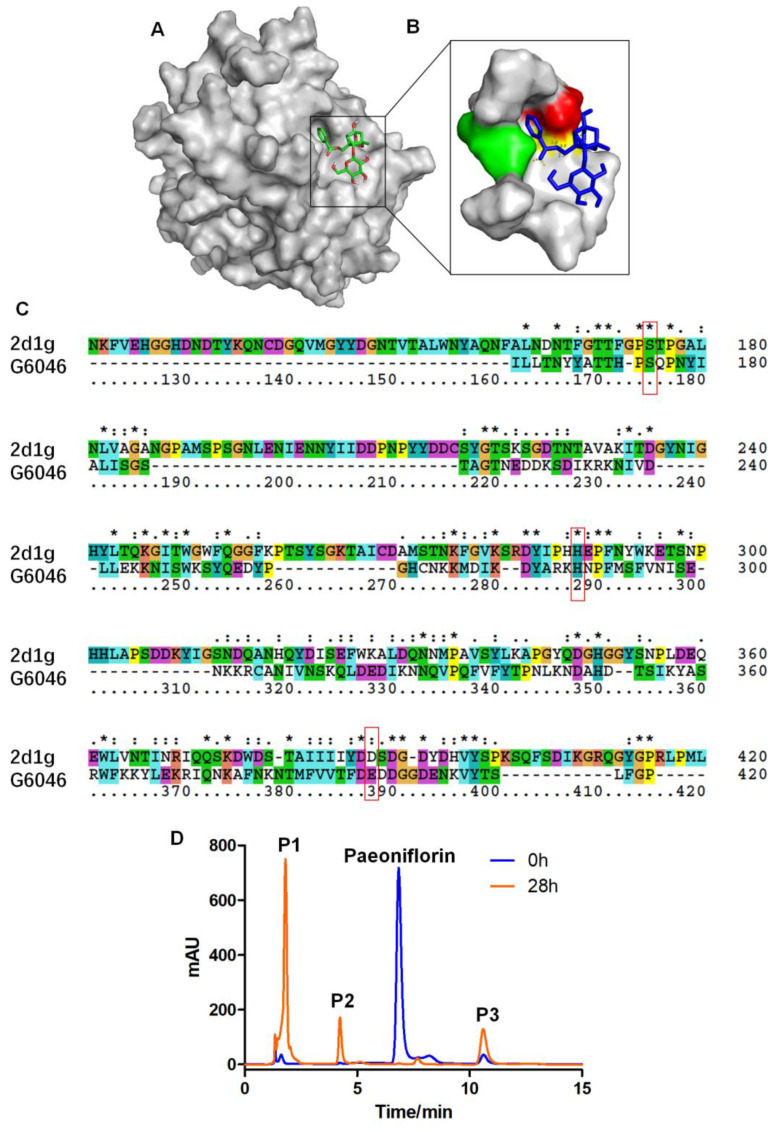
Transformation of paeoniflorin to P1, P2, and P3 mediated by G6046. (**A**) Molecule docking between G6046 and paeoniflorin produced using PSIPRED 4.0 and Phyre2; (**B**) surface topography of the active site entrance, Ser-87 is in red, His-150 is in green, and Glu-235 is in yellow; (**C**) sequence alignment of ApcA 2d1g and G6046 Ser-87, His-150, and Glu-235 in G6046 corresponding to Ser-175, His-288, and Asp-388 in ApcA 2d1g are indicated in the red boxes; (**D**) HPLC analysis of the conversion activity of G6046, obtained on an Agilent ZORBAX SB-C18 (4.6 × 250 mm, 5 μm); column temperature, 30 °C; mobile phase, H_2_O (0.2% phosphoric acid):methanol = 70:30 (*v*/*v*); flow rate, 1.0 mL/min; injection volume, 10 μL; detection wavelength, 230 nm. After 28 h of reaction, the main conversion products were labelled P1, P2, and P3. * indicates positions which have a single, fully conserved residue, : indicates that one of the following “strong” groups is fully conserved.

The secondary and tertiary structures of G6046 were predicted using PSIPRED 4.0 and Phyre2, followed by computer-aided docking analysis. The model of G6046 was built based on chain B of Acid phosphatase A (ApcA, PDB code: 2d1g [26]), which had a match confidence of 100% and 28% sequence identity. Figure 6A shows the virtual docking principle between paeoniflorin and G6046. When paeoniflorin was in contact with G6046, the two molecules exhibited strong docking capabilities with a binding free energy of −3.25 kcal/mol. Figure 6B shows a catalytic triad formed by Ser-87, His-150, and Glu-235, in which three hydrogen bonds are formed between glutamate and the benzoate bonds in paeoniflorin. It was speculated that the grooved part, which is close to 7 fold domains, may be the activity center of G6046 since it strongly matches the activity domain in 2d1g (Figure 6C). Therefore, G6046 may be a phosphatase or phosphoesterase. 

High performance liquid chromatography (HPLC) analysis was performed to investigate the paeoniflorin-converting activity of G6046-NΔ82-22b. HPLC analysis of G6046-NΔ82-22b found it was able to convert paeoniflorin into three new substances, (Figure 6D) P1, P2, and P3. The G6046 enzyme expressed and purified in *E. coli* has the same activity and exerts the same conversion on paeoniflorin as the induced exoenzyme of *Cunninghamella blakesleeana* (Figure 1B and Figure 6D). Enzyme activity was determined at pH 9, 45 °C, with the specific enzyme activity of 14.56 U/mg. 

### 2.7. Selection of Solvent System and CCC Separation

Several prerequisites should be considered. Partition coefficients of different solvent systems in the range of 0.5–2 are important for successful separation by CCC [27]. Moreover, the separation factor between two constituents (α = *K*_1_/*K*_2_, *K*_1_ > *K*_2_) is at least 1.5. Solubility and stability of the analytes in solvent should be considered at the same time, as is the same case in the rapid and clear separation of solvent system into phases. It has been reported that paeoniflorin had been separated by an ethyl acetate/n-butanol/water (3/2.5/5, *v*/*v*) solvent system [28]. We found that the P2 product obtained by using the ethyl acetate/n-butanol/water (3/2.5/5, *v*/*v*) solvent system may contain unconverted paeoniflorin. However, our optimization, based on the determination of the *K* and α values of the convert products, as shown in Table 2, revealed that ethyl acetate/n-butanol/water (1/4/5, *v*/*v*) could be a potential solvent system to produce a satisfactory separation. 

**Figure 7:**
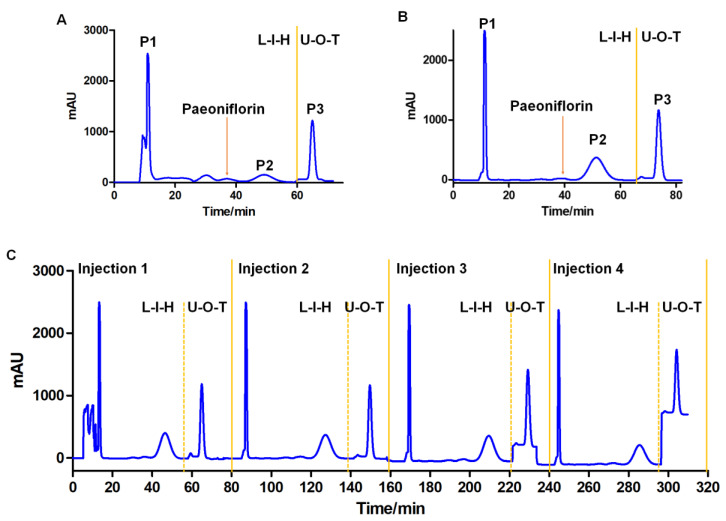
HPCCC separation of the paeoniflorin conversion products from analytical (**A**) to semi-preparative (**B**) to continuous injection in semi-preparative (**C**). (**A**) Analytical scale: sample, 30 mg/0.5 mL; column, Spectrum HPCCC-22.5 mL; flow rate, 1.0 mL/min; rotational speed, 250 g; retention of the stationary phase, 58.4%; (**B**) Semi-preparative scale: sample, 97 mg/2 mL; Spectrum HPCCC-133.5 mL; flow rate, 4.0 mL/min; rotational speed, 250 g; retention of the stationary phase, 56.7%; (**C**) Continuous injection in semi-preparative; other conditions are the same for (**A**–**C**), elution time: 60 min for L-I-H mode (reversed phase elution) and 20 min for U-O-T mode (normal phase elution); detection wavelength, 230 nm.

**Figure 8:**
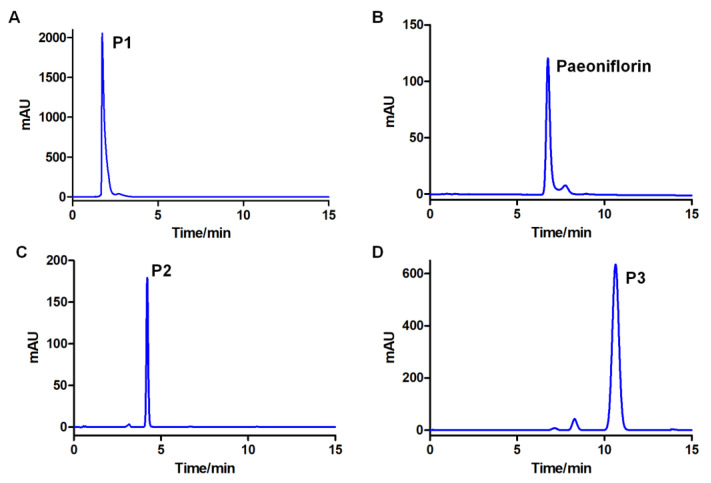
HPLC-DAD chromatograms of paeoniflorin conversion products. Conditions are the same as in Figure 6. (**A**–**D**) are DAD chromatograms of P1, paeoniflorin, P2, and P3, respectively. The purity of P2 was 99.81%, and the purity of P3 was 95.39%.

Therefore, 1/4/5 ethyl acetate/n-butanol/water was used to separate the components converted by G6046 on an HPCCC instrument. Based on P3′s high K values, most of it was likely partitioned to the upper phase and was hard to elute in the lower phase. The CCC elution was operated in L-I-H [29] (lower phase as mobile phase, eluted from inner head) mode followed by the upper phase as mobile phase in the tail-to-head mode (U-O-T). Figure 7A,B illustrates the CCC separation of the conversion products from analytical to semi-preparative scale. As the system equilibrium was maintained during the two-mode elution process, the repetitive injection of the sample can be performed right after one run. Four injections were performed over 320 min (Figure 7C). For semi-preparative separation, 97 mg of the sample was loaded onto a 133.5 mL column and the three peak fractions could be baseline separated (Figure 7B). The purity of each fraction corresponding to each chromatographic peak was analyzed by HPLC. The results showed that the diode array detection (DAD)-purity of P2 was 99.81%, and the purity of P3 was 95.39% (Figure 8). After concentration and drying, 8.7 mL of P1 as an oily liquid, and 25.23 mg of P2 and 34.17 mg of P3 as amorphous white powder were obtained.

### 2.8. UPLC-Q-TOF-MS Analysis of the Conversion Products 

**Figure 9:**
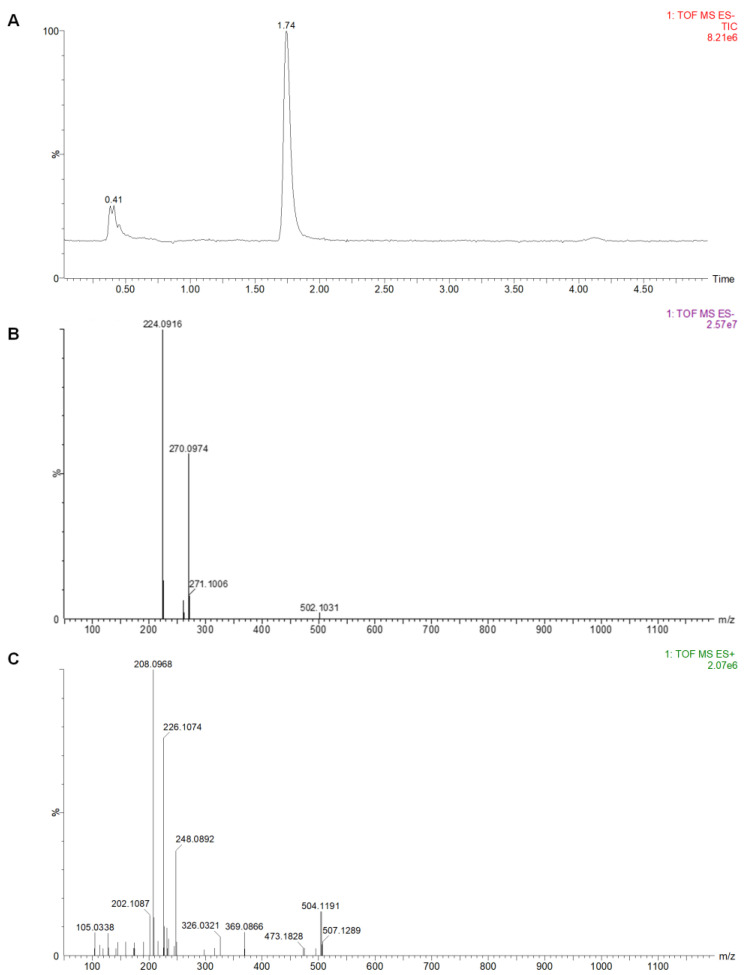
The mass spectra of component P2. (**A**) is the total ion flow diagram; (**B**,**C**) are the primary mass spectrum in negative and positive ion modes.

After HPCCC separation, ultra-performance liquid chromatography coupled with quadrupole time-of-flight mass spectrometry (UPLC-Q/TOF-MS) was used to identify the P1, P2, and P3. Although the DAD-purity of P1 was high, the mass spectrometry results indicated that it was a mixture. P1 was not investigated for identification, given that it was a mixture of compounds (Figure S5). As shown in Figure 9B, the compound P2 showed in its ESI^−^ mass spectra ion fragments at *m*/*z* 224.0916 and *m*/*z* 270.0974 that were assigned to molecular ion [M-H]^−^ and [M-H+HCOOH]^−^, respectively. Additionally, the positive ion fragments at *m*/*z* 248.0892, *m*/*z* 226.1074, and *m*/*z* 208.0968 were assigned to [M+Na]^+^, [M+H]^+^, and [M+H-H_2_O]^+^, respectively, as shown in Figure 9C. Therefore, P2 was determined to have a molecular formula of C_11_H_15_O_4_N, with a molecular weight of 225.

**Figure 10:**
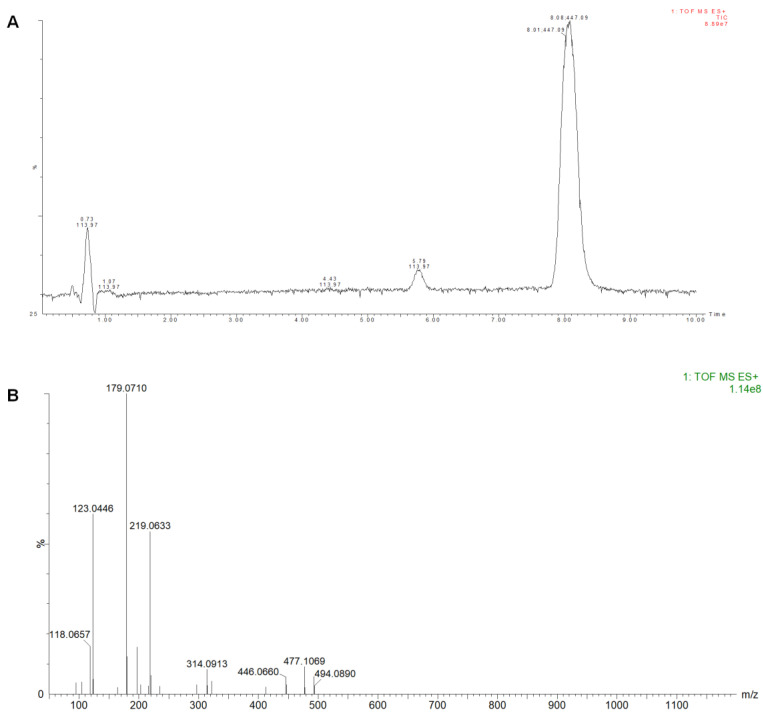
The mass spectra of component P3. (**A**) is the total ion chromatogram; (**B**) is the primary mass spectrum in positive ion mode.

The mass spectra of component P3 can be seen in Figure 10. Its ESI^+^ mass spectra ion fragments at *m*/*z* 219.0628, *m*/*z* 179.0709, and *m*/*z* 123.0444 were assigned to molecular ion [M+Na]^+^, [M+H-H_2_O]^+^, and [M+H-C_3_H_4_O-H_2_O]^+^, respectively. The ion fragment at *m*/*z* 123 was assigned to benzoic acid after a loss of glycerin. The mass pattern showed a loss of glycerin from *m*/*z* 197 to *m*/*z* 123, corresponding to C_3_H_6_O_2_ group elimination. Therefore, P3 was determined to have a molecular formula of C_10_H_12_O_4_ with a molecular weight of 196. Almost no signal was observed in negative ion mode.

### 2.9. Structural Identification by NMR 

**Figure 11:**
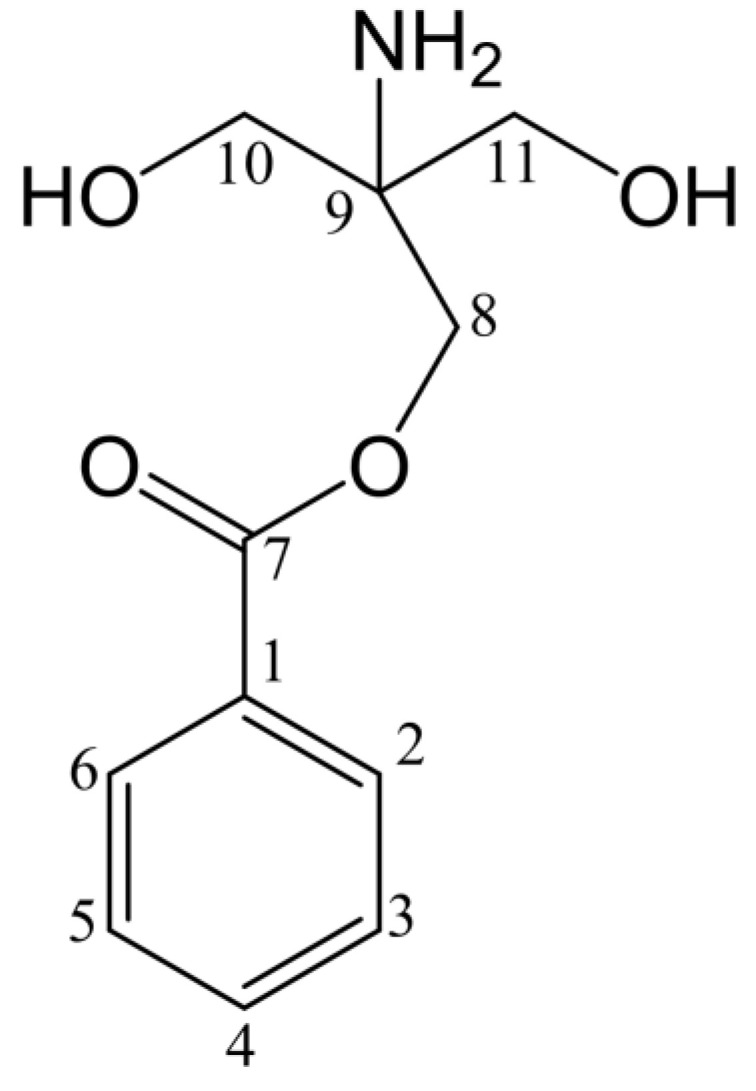
Structure of P2.

The structure of component P2 was determined using ^13^C NMR spectrum (Figure S1), and ^1^H NMR spectrum (Figure S2). The buffer used to purify G6046 contains Tris (C_4_H_11_NO_3_), so the reaction system contained Tris. ^13^C NMR δC 135.64 (CH), 131.57 (C), 128.63 (CH), 127.72 (CH) and ^1^H NMR δH 7.77 (m, 2H), 7.50 (t, 1H), 7.44 (t, 2H) show the presence of monosubstituted benzene ring fragments in compound P2. With the catalysis of G6046, the paeoniflorin was converted to benzoic acid fragment and then the carboxyl group on the benzoic acid fragment reacted with the hydroxyl group on Tris (See Table 3). Accordingly, we determined the structural formula of P2 to be 2-amino-2-p-hydroxymethyl-methyl alcohol benzoate (Figure 11). 

**Figure 12:**
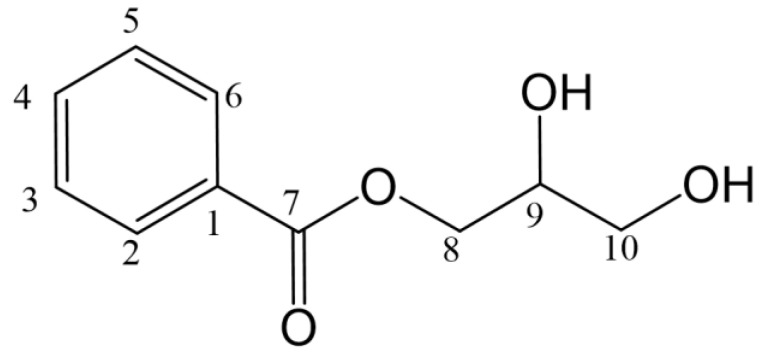
Structure of P3.

The ^13^C NMR spectrum and ^1^H NMR spectrum of component P3 are shown in Figures S3 and S4. The corresponding relationship between hydrocarbons is shown in Table 4. ^13^C NMR δC 133.64 (CH), 130.38 (C), 129.64 (CH), 129.09 (CH) and ^1^H NMR δH 8.00 (d, 2H), 7.66 (t, 1H), 7.53 (t, 2H) indicated that P3 also has a monosubstituted benzene ring fragment. ^13^C NMR δC 69.82 (CH), 66.82 (CH_2_), 63.08 (CH_2_) and ^1^H NMR δH 4.18 (dd, 1H), 4.31 (dd, 1H), 3.80 (q, 1H), 3.45 (hept, 2H) indicates that the compound also contains two methylene groups connected with oxygen-containing groups and one methyne, which was analyzed as glycerol structure. In addition, δC 166.24 was a carbonyl signal. Combined with the above mass spectrum analysis, it can be determined that P3 is glycerin-1-benzoatel benzoate (Figure 12). The NMR data for glycerin-1-benzoatel benzoate are consistent with the data reported in the literature [30]. 

## 3. Discussion

In this study, the gene sequence of a paeoniflorin-converting enzyme from *Cunninghamella blakesleeana* (AS 3.970) was identified via transcriptomics and designated G6046. The conversion activity of the expressed protein was verified in vitro, and the conversion products were separated and purified by high performance counter-current chromatography (HPCCC). Three component fractions were observed—P1, P2, and P3—and 25.23 mg and 34.17 mg of P2 and P3 with the HPLC purity of 99.81% and 95.39%, respectively, were obtained. Using UPLC-Q-TOF-MS and NMR analysis, they were determined to be 2-amino-2-p-hydroxymethyl-methyl alcohol-benzoate (tris-benzoate) and 1-benzoyloxy-2,3-propanediol (1-benzoyloxypropane-2,3-diol). As shown in Figure S5, we found that the rest of the molecule that lost benzoic moiety is debenzoylpaeoniflorin in P1 fraction, which has the molecular formula of C_16_H_24_O_10_. Its ESI^−^ mass spectra ion fragments at *m*/*z* 375.1304 and *m*/*z* 751.2679 were assigned to molecular ion [M-H]^−^ and [2M-H]^−^. We have also done MS analysis of the productions by the induced exoenzyme (Figure 1B). The MS results were the same as those after we purified P2 and P3. 

There are many studies on the pharmacokinetics of paeoniflorin [15,18,31,32,33]. Previously, many metabolites of paeoniflorin (such as 7R-paeonimetabolin I, 7S-paeonimetabolin I, 7R-paeonimetabolin II, 7S-paeonimetabolinII, albiflorin, 4-O-methyldebenzoylpaeoniflorin, desdimethyl-oxidationpinane glucuronide, and paeoniflorgenin) had been found, and some of them were produced by intestinal bacteria [34,35,36,37]. As mentioned above, the bioavailability of paeoniflorin is very low after oral administration, so it is suspected that the pharmacological action of paeoniflorin may be produced by its metabolites of intestinal bacteria [14,20]. However, there are few research studies on benzoic acid as its metabolite [14,16]. Nowadays, benzoic acid is a commonly used preservative and it is also an important chemical raw material, which can be used to prepare sodium benzoate preservatives and to synthesize drugs and dyes. When we use “benzoic acid” and “antidepressant” to search references, there are few hits that associate benzoic acid with antidepressant, but more and more studies show that benzoate has antidepressant effects [38,39,40,41]. Sodium benzoate, an FDA-approved drug against urea cycle disorders in children [42], is widely used as a food additive, which is long known for its microbicidal effect. It alters the neuroimmunology of experimental allergic encephalomyelitis (EAE) and ameliorates the disease process of EAE [43,44]. Sodium benzoate can increase the brain-derived neurotrophic factor (BDNF) expression in neurons in a dose-dependent and time-dependent manner [45,46]. in vitro and in vivo studies have indicated that benzoate exerts anti-inflammatory effects by inhibiting microglial activity. The benzoate acts as both an N-methyl-d-aspartate glutamate receptor (NMDAR) modulator and an anti-inflammatory drug, and, thus, can be effective in the treatment of depression [40,47,48].

**Figure 13:**
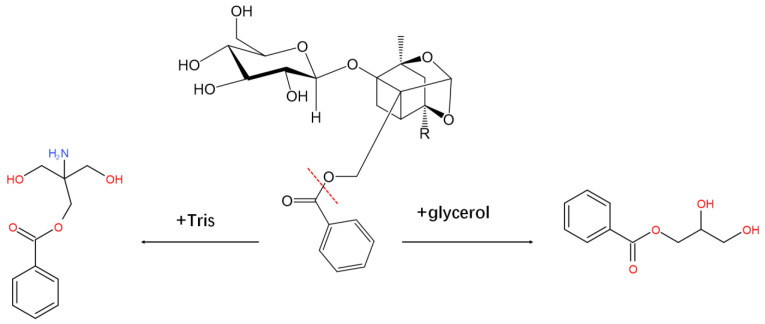
The structure analysis of the product formation process. The benzoic acid group was enzymatically hydrolyzed by G6046. The cleaved benzoate reacted with either Tris or glycerol presented in the experimental buffer to produce P2 and P3.

According to the structure analysis of the products, both products contain benzoate groups. As shown in Figure 13, in the presence G6046, the benzoate bond in paeoniflorin was broken, and the benzoic acid group was enzymatically hydrolyzed. P2 and P3 resulted from the cleaved benzoate reacting with either Tris or glycerol present in the experimental buffer. On this basis, we speculate that benzoic acid is a characteristic gut microbial metabolite of paeoniflorin and may metabolize to produce new benzoate compounds. Sodium benzoate was also a metabolite of cinnamon [49]. Some other benzoate compounds are promising antidepressant candidates, such as 4-[1-[1-(benzoyloxy)cyclohexyl]-2-(dimethylamino)ethyl]-phenyl benzoate [50], 4-[2-(dimethylamino)-1-(1-hydroxycyclohexyl)-ethyl]-phenyl benzoate hydrochloride [51], and potassium 2-(1-hydroxypentyl)-benzoate [52]. As described above in the introduction, the degradation by gut microbiota enzymes may be one of the main reasons for the low bioavailability of paeoniflorin, and the pharmacological effect of paeoniflorin may be produced by the resultant metabolites. The clinical applicability of paeoniflorin is greatly limited due to its low bioavailability and unrevealed pharmacological mechanism. Our study directly confirmed that one of the eukaryotic microorganisms can metabolize paeoniflorin. The study of paeoniflorin metabolites and their intestinal bacterial metabolism is therefore of great significance for an in-depth understanding of its pharmacodynamic mechanisms. Benzoic acid or these newly generated benzoate substances may be more easily absorbed into the blood by the gut, and more easily penetrate the blood–brain barrier, enter the central nervous system, and play a depressive pharmacological role.

## 4. Materials and Methods

### 4.1. Strains and Culture Medium

*Escherichia coli* BL21 (DE3) and BL21 TOP10 strains were purchased from TransGen Biotech (Beijing, China). *Cunninghamella blakesleeana* (AS 3.970) was purchased from the Chinese Academy of Sciences Institute of Microbiology. Luria-Bertani (LB) (10 g/L tryptone, 10 g/L NaCl and 5 g/L yeast extract) medium was used for all *Escherichia coli* strains. Potato Dextrose Agar (PDA) medium and Potato Dextrose Broth (PDB) were used for culturing *Cunninghamella blakesleeana* (AS 3.970).

### 4.2. Reagents and Materials

DNA restriction enzymes, DNA Ligation high, Taq DNA polymerase, DNA marker, and protein marker were purchased from the Beyotime Institiute of Biotechnology (Shanghai, China). The Gel Extraction kit and Plasmid Miniprep kit were purchased from TIANGEN biotech (Beijing) Co., Ltd., (Beijing, China). Ni Sepharose™ 6 Fast Flow was purchased from GE Healthcare (Fairfield, MA, USA). 

Ethyl acetate, n-butanol, and methanol were bought from Macklin (AR, Shanghai Macklin Biochemical Co. Ltd., Shanghai, China). NaCl, HCl, NaOH (AR, China National Pharmaceutical Group Corporation, Beijing, China), and distilled water were used. Chromatographic-grade methanol from Fisher (HPLC, Fisher Scientific, Waltham, MA, USA) was used for high performance liquid chromatography (HPLC) analysis. Paeoniflorin standards were purchased from YuanYe company (Shanghai, China). 

### 4.3. Biotransformation by Cunninghamella blakesleeana

The bacterial powder of *Cunninghamella blakesleeana* (AS 3.970) was diluted in 5 mL of sterile water, inoculated in PDA plate culture medium, and incubated at 28 ºC. When the mycelium grew on the plate, they were transferred to a new PDA plate. Vibrant bacteria were obtained after three rounds of activation. The activated bacterial cells were incubated at 28 °C for about seven days until the mycelium grew vigorously and spores were abundant. The spores on the plate were washed into a sterile triangle bottle with 5 mL of sterile water to make spore fluid [53]. The number of spores in the spore fluid was counted by hemocytometry to determine the inoculation amount. 

The spore fluid was inoculated into the liquid medium at a rate of 3% and then incubated at 28 °C for 48 h. A total of 100 mL of the *Cunninghamella blakesleeana* (AS 3.970) medium was poured off and induced by adding 1.5 mL of paeoniflorin solution (1 mg/mL). The concentration of paeoniflorin in the medium was continuously monitored via HPLC for 5 days. After the incubation, the medium was centrifuged at 16,000× *g*, 4 °C for 30 min to obtain the supernatant. The paeoniflorin was identified by comparing the retention times with that of the standard sample.

### 4.4. Identification of Proteins in Culture Medium after Paeoniflorin Induction 

The paeoniflorin-induced supernatant medium and the paeoniflorin-free supernatant medium (used as control) were passed through the 0.22 μm microporous membrane to remove bacterial interference. The proteins in both supernatants were precipitated by adding ammonium sulfate while the sample was cooled in an ice bath. Ammonium sulfate was slowly added into the supernatant with stirring until saturation and the protein precipitation was collected by centrifugation (16,000× *g*, 4 °C, 30 min). The protein precipitates were dissolved in 10 mL of PBS and the undissolved substances were pelleted via centrifugation (16,000× *g*, 4 °C, 30 min). The supernatant was concentrated in a 10 kD cutoff filter tube to 200 μL. Exoenzymes in both paeoniflorin-induced and paeoniflorin-free supernatants were analyzed by SDS-PAGE. After Coomassie Brilliant Blue staining, the gels were observed through a gel imaging system. The relevant protein bands in the SDS-PAGE were digested with trypsin. Briefly, the gels were cut into small pieces, which were then washed with 200 μL of distilled water and destained with 200 μL destaining solution (30 mM K_3_Fe(CN)_6_:100 mM NaS_2_O_3_, 1:1, *v*/*v*). After being dehydrated with ACN, the proteins in gel were reduced with 20 mM DTT at 56 °C for 1 h and then alkylated with 40 mM iodoacetamide (IAM) at room temperature in the dark. The proteins were then digested with trypsin at 37 °C overnight. The digestion was stopped by adding formic acid to a final concentration of 0.5%. The tryptic peptides were extracted by can, and the resulting peptide mixtures were dried and stored at −80 °C for further MS analysis. The protein mass spectrometry experiments were performed with a linear ion trap mass spectrometer (LTQ, Thermo Fisher Corporation, San Jose, CA, USA) that coupled with the Thermo Finnigan Surveyor nano HPLC system in the Center for Biological Imaging (CBI), Institute of Biophysics, Chinese Academy of Sciences. MS data were analyzed using Proteome Discoverer 1.4.1.14 via the Sequest HT search engine. Given the lack of a reference proteome, the peptide sequences were then compared against the non-redundant (nr) protein database at the National Center for Biotechnology Information (NCBI) using the Basic Local Alignment Search Tool (BLAST). 

### 4.5. Transcriptomic Analysis 

AS 3.970 from the plate medium was inoculated into eight flasks with liquid medium, which were randomly divided into two groups. One of the groups was induced by adding 1.5 mL of paeoniflorin solution (1 mg/mL) after incubating for 48 h, then the bacterial media was incubated continuously for 24 h. The bacteria were harvested by centrifugation (16,000× *g*, 4 °C, 30 min). AS 3.970 bacteria in each flask were immediately frozen in liquid nitrogen and stored at −80 °C. Exoenzymes in the supernatant were isolated as described above and analyzed using SDS-PAGE. According to the results of SDS-PAGE, three samples with relatively consistent bands of each group were selected, and the corresponding frozen bacterial cell pellets were sent for RNA-Seq testing [54]. The samples treated with paeoniflorin solution were recorded as I1, I2, and I3, and those without paeoniflorin solution attack were recorded as U1, U2, and U3. The protein MS results of the paeoniflorin-induced bands and transcriptomics results of the up-regulated genes after paeoniflorin induction were compared. 

### 4.6. Construction of the G6046 Expression Vectors 

According to the comparative analysis, the gene with GeneID of G6046_c0_g1 may encode the key enzyme for paeoniflorin transformation in AS3.970. The resultant sequences were submitted to GenBank to obtain accession numbers (OP856858). The molecular weights and theoretical pI of G6046 were calculated using the ExPASy program [55]. The transmembrane regions, signal peptides, secondary structure prediction, and tertiary protein modeling, prediction, and analysis of G6046 were obtained using the PSIPRED 4.0 [56] and Phyre2 web [57]. 

The G6046 gene was synthesized by RuiBiotech Inc. (Beijing, China). To express G6046 in an *E. coli* expression system, the gene encoding G6046 was cloned into pET-28b and pET-22b vectors (Novagen-EMD Millipore) using standard cloning methods. Meanwhile, G6046 truncations were constructed to optimize gene expression in *E. coli*. The details of the clones for G6046 and its truncations can be seen in Table 1. The recombinant vectors were transformed into TOP10 cells for plasmid construction and maintenance. After culturing on the agar LB medium plate overnight at 37 °C, a single clone was picked up and cultivated in 5 mL LB media (100 μg/mL kanamycin or 100 μg/mL ampicillin) at 37 °C for 12 h. Plasmids were extracted using the Plasmid Miniprep kit, which was purchased from Solarbio (Beijing, China). The sequences of the wild-type and all truncations were confirmed by DNA sequencing. 

### 4.7. Expression and Purification of G6046 and Truncations in E. coli BL21 (DE3)

*E. coli* BL21 (DE3) strain (TransGen Biotech, Beijing, China) was used for protein expression [58]. The plasmids of G6046 or its truncations were transformed into *E. coli* BL21 (DE3) strain, and the single clone was amplified to 1 L LB medium (100 μg/mL kanamycin or 100 μg/mL ampicillin). The cells were cultured at 37 °C and IPTG was added to a final concentration of 0.5 mM when OD_600_ = 0.8. Cells were incubated for another 20 h at 16 °C. The cells were harvested by centrifugation and stored at −40 °C for further use.

The recombinant G6046 protein was purified using the affinity chromatography method. The cells were re-suspended in the lysis buffer (50 mM Tris, pH 7.5, 100 mM NaCl, 10% glycerol) and then disrupted by sonication for 30 min (5 s on, 10 s off) on ice. The cell lysate was centrifuged at 16,000× *g* for 1 h. Two milliliters of Ni-NTA resin was added to a gravity column and equilibrated with the lysis buffer. The supernatant was loaded onto the column and washed with 50 mL of the lysis buffer containing 20 mM imidazole. The protein was eluted with 20 mL of the lysis buffer supplemented with 200 mM imidazole. The concentration of eluted G6046 protein was measured using the Bradford assay (TIANGEN biotech Beijing Co., Ltd., Beijing, China) and the purity was evaluated by SDS-PAGE.

### 4.8. HPLC Analysis of the Biotransformation Activity 

A total of 1 mg of paeoniflorin was dissolved in 960 μL of buffer (50 mM Tris, 100 mM NaCl, 10% glycerol, pH 8.5) in a 2 mL centrifuge tube. After dissolving, 40 μL of the pure G6046 protein solution with a concentration of 1 mg/mL was added to monitor the reaction for 96 h. The concentration of paeoniflorin and the conversion products were detected by HPLC using an Agilent ZORBAX SB-C18 (250 × 4.6 mm i.d., 5 μm). The HPLC mobile phases were water (0.2% phosphoric acid (A) and methanol (B) (A:B = 70:30). The conditions were as follows: flow rate, 1.0 mL/min; column temperature, 30 °C; detection wavelength, 230 nm; injection volume, 10 μL. 

The enzymatic activity of G6046 was defined as the amount of enzyme required to convert 1 μmol of paeoniflorin in 1 h at 25 °C. 

### 4.9. HPCCC Separation of the Converting Products

The CCC apparatus used in this study was a Spectrum HPCCC (Dynamic Extraction, UK). The Spectrum HPCCC contains an analytical column (0.8 mm i.d. tubing, 22.5 mL) and a semi-preparative column (1.6 mm i.d., 133.5 mL) with rotation speeds adjustable from 0 to 250 g. The column temperature was controlled by a SH150-1500 constant temperature regulator (Lab Tec, Beijing, China). A KNAUER Smartline HPLC system (Berlin, Germany) containing two p-1000 pumps, a UV-2500 detector, and a EuroChrom workstation was equipped with the Spectrum HPCCC.

The selection of a two-phase solvent system for CCC is based on the partition coefficient (*K*) and separation factors (α) of each convert product. The *K* value of each component was measured by HPLC. Generally, 10 mg of crude sample was dissolved in the selected two-phase solvents (Table 2) with 1 mL of upper phase and 1 mL of lower phase. After violently shaking and equilibrating the two phases completely, 0.5 mL solution of each phase was taken and analyzed by HPLC. The *K* values of each target component were calculated from the peak area obtained from the upper phase divided by that of the lower phase. Separation factors (α) represented the ratio of the *K* values of two adjacent target components.

The multilayer column was first filled with the upper phase (stationary phase), and the mobile phase was then pumped into the column at the flow rate of 4 mL/min while the apparatus was rotated at a speed of 250 g. The flow rate of the mobile phase was 1.0 mL/min on the 22.5 mL analytical column and 4.0 mL/min on the 133.5 mL semi-preparative column of the Spectrum HPCCC. After equilibration, the 30 mg in 0.5 mL of the sample dissolved in the upper phase was injected into the analytical column. The sample size was then scaled up to 97 mg in 2 mL to inject onto the semi-preparative column. The dual-mode elution strategy [59] was employed in the separation due to the high partition coefficient values of the P3. The column was first eluted with the lower phase as mobile phase in head-to-tail mode (L-I-H) for 60–65 min, followed with the upper phase as mobile phase in the tail-to-head mode (U-O-T). Continuous injection was also done on the semi-preparative column. Chromatograms were recorded at 230 nm and the peak fractions were collected according to the chromatograms. The purities of the separated targets were analyzed by HPLC.

### 4.10. Identification of the Separates by UPLC-Q-TOF-MS and NMR 

The paeoniflorin and conversion products were diluted in HPLC-grade methanol to a final concentration of 1 mg/mL and then separated and identified using the Waters Acquity UPLC system and the Waters Q-ToF Premier mass spectrometer using an ACQUITY UPLC BEH C18 analytical column (i.d. 2.1 × 50 mm, 1.9 mm) [60,61]. The UPLC mobile phases were water (0.2% phosphoric acid) (A) and methanol (B) (A:B = 70:30). The sample and the column were kept at 40 °C, and 0.2 μL of 1 mg/mL sample was injected into the column for each analysis.

The paeoniflorin and conversion products were analyzed on the Q-TOF-MS (Waters, Milford, MA, USA). Mass spectra were acquired using an electrospray ionization (ESI) source in positive and negative mode for the identification and quantification. The flow rates of the cone gas and the desolvation gas were set at 50 and 800 L/h. Source temperature, the capillary voltage, and the sampling cone voltage were 350 °C, 2.5 kV, and 40 V, respectively. A range of *m*/*z* 50–1200 for 0.5 s was needed to achieve full scan spectra. LockSpray reference ions (554.2615, [M-H]^−^) were infused during data acquisition for online calibration to ensure mass accuracy. The data were analyzed using Mass-Lynx software (Waters Corp., Manchester, UK).

The NMR data were obtained on an Aglient DD2 600 MHz NMR spectrometer (Agilent, Santa Clara, CA, USA) with tetramethylsilane as an internal standard. SpectraMax 190 (Molecular Devices, Sunnyvale, CA, USA) ELISA was used in this study. ^13^C NMR (150 MHz, DMSO-*d*_6_) δ [ppm] of P2:167.76 (-O-**C**=O), 135.64 (ArC), 131.57 (ArC), 128.63 (ArC), 127.72 (ArC), 72.94 (-O-**C**H_2_-C-), 63.56 (HO-CH_2_-), 63.08 (HO-CH_2_-), 60.99 (-C-); ^1^H NMR (600 MHz, DMSO-*d*_6_) δ [ppm]: 7.80–7.75 (m, 2H), 7.50 (t, *J* = 7.3 Hz, 1H), 7.44 (t, *J* = 7.5 Hz, 2H), 3.67 (s, 6H), 3.44–3.24 (m, 4H). ^13^C NMR (150 MHz, DMSO-*d*_6_) δ [ppm] of P3: 166.24 (-O-C=O), 133.64 (ArC), 130.38 (ArC), 129.64 (ArC), 129.09 (ArC), 69.82 (HO-CH_1_-), 66.82 (-O-CH_2_-C-), 63.08 (HO-CH_2_-). ^1^H NMR (600 MHz, DMSO-*d*_6_) δδ [ppm] of P3: 8.00 (d, *J* = 7.7 Hz, 2H), 7.66 (t, *J* = 7.4 Hz, 1H), 7.53 (t, *J* = 7.6 Hz, 2H), 4.31 (dd, *J* = 11.1, 4.0 Hz, 1H), 4.18 (dd, *J* = 11.1, 6.3 Hz, 1H), 3.80 (q, *J* = 5.4 Hz, 1H), 3.45 (hept, *J* = 5.8 Hz, 2H). 

### 4.11. Paeoniflorin AutoDock Vina Docking Analysis

Analysis of paeoniflorin docking into G6046 was conducted using AutoDockTools software (v4.2). The tertiary protein model of G6046 was obtained from Phyre2 web [58]. During the docking process, the protein was kept rigid, while the ligands were treated as fully flexible. Parameters were set to the default values.

## Figures and Tables

**Table 1:** Constructs of G6046.

Clone	Vector	Restriction Site			Cell Strain	Expression
G6046-22b	pET22b	*Nde*I		*Xho*I	BL21 (DE3)	Inclusion body
G6046-28b	pET28b	*Nde*I		*Xho*I	BL21 (DE3)	Inclusion body
MBP-G6046	pET28b	*Nco*I	*Nde*I	*Xho*I	BL21 (DE3)	Inclusion body
T4L-G6046	pET28b	*Nco*I	*BamH*I	*Xho*I	BL21 (DE3)	Inclusion body
G6046-NΔ57-22b	pET22b	*Nde*I		*Xho*I	BL21 (DE3)	Inclusion body
G6046-NΔ57-28b	pET28b	*Nde*I		*Xho*I	BL21 (DE3)	Inclusion body
MBP-G6046-NΔ57	pET28b	*Nco*I	*Nde*I	*Xho*I	BL21 (DE3)	Inclusion body
T4L-G6046-NΔ57	pET28b	*Nco*I	*BamH*I	*Xho*I	BL21 (DE3)	Inclusion body
G6046-NΔ82-22b	pET22b	*Nde*I		*Xho*I	BL21 (DE3)	√
G6046-NΔ82-28b	pET28b	*Nde*I		*Xho*I	BL21 (DE3)	Inclusion body
MBP-G6046-NΔ82	pET28b	*Nco*I	*Nde*I	*Xho*I	BL21 (DE3)	Inclusion body
T4L-G6046-NΔ82	pET28b	*Nco*I	*BamH*I	*Xho*I	BL21 (DE3)	Inclusion body

**Table 2:** Partition coefficients (K) and separation factors (α) of the four components in four solvent systems.

Solvent System Ethyl Acetate/n-Butanol/Water (*v*/*v*)	P1 (*K*_1_)	Paeoniflorin	P2 (*K*_2_)	P3 (*K*_3_)	α	
		(*K*_p_)			*K*_p_/*K*_1_	*K*_2_/*K*_p_
3/2.5/5	0.35	0.68	0.73	2.76	1.94	1.07
3/2/5	0.24	0.54	0.59	2.94	2.25	1.09
2/3/5	0.43	1.03	1.18	3.52	2.39	1.14
1/4/5	0.68	1.22	1.89	7.35	1.79	1.54

**Table 3:** ^13^C NMR and ^1^H NMR data for P2.

No.	δC (*J* in Hz)	δH (*J* in Hz)
1	131.57	
2	128.63	7.77 (m, 2H)
3	127.72	7.44 (t, *J* = 7.5 Hz, 2H)
4	135.64	7.50 (t, *J* = 7.3 Hz, 1H)
5	127.72	7.44 (t, *J* = 7.5 Hz, 2H)
6	128.63	7.77 (m, 2H)
7	167.76	
8	72.94	3.67 (s, 2H)
9	60.99	
10	63.08	3.67 (s, 2H)
11	63.56	3.67 (s, 2H)

**Table 4:** ^13^C NMR and ^1^H **NMR** data for P3.

No.	δC (*J* in Hz)	δH (*J* in Hz)
1	130.38	
2	129.64	8.00 (d, *J* = 7.7 Hz, 2H)
3	129.09	7.53 (t, *J* = 7.6 Hz, 2H)
4	133.64	7.66 (t, *J* = 7.4 Hz, 1H)
5	129.09	7.53 (t, *J* = 7.6 Hz, 2H)
6	129.64	8.00 (d, *J* = 7.7 Hz, 2H)
7	166.24	
8	66.82	4.18 (dd, *J* = 11.1, 4.0 Hz, 1H)
		4.31 (dd, *J* = 11.1, 6.3 Hz, 1H)
9	69.79	3.80 (q, *J* = 5.4 Hz, 1H)
10	63.08	3.45 (hept, *J* = 5.8 Hz, 2H)

## Data Availability

The datasets of this study are available from the corresponding author on reasonable request.

## Supplementary Materials

The following supporting information can be downloaded at: https://www.mdpi.com/article/10.3390/molecules28031289/s1, Figure S1: The ^13^C NMR spectra of P2 (151 MHz, DMSO-*d*_6_).; Figure S2: The ^1^H NMR spectra of P2 (600 MHz, DMSO-*d*_6_); Figure S3: The ^13^C NMR spectra of P3 (151 MHz, DMSO-*d*_6_); Figure S4: The ^1^H NMR spectra of P2 (600 MHz, DMSO-*d*_6_); Figure S5: The mass spectra of component P1; Table S1: MS analysis Results of B1; Table S2: MS analysis Results of B2; Table S3: MS analysis Results of B3; Table S4: Statistics of data output; Table S5: Statistics of differential gene number.
