# Supplementary material for: The Study of a Novel Paeoniflorin-Converting Enzyme from Cunninghamella blakesleeana
Source: Molecules. 2023 Jan 29;28(3):1289. doi: 10.3390/molecules28031289 (PMC9921665; doi:10.3390/molecules28031289)
Supplement: Supplementary file 1 [file molecules-28-01289-s001.zip › molecules-2136310-supplementary.pdf]

Supplementary Information

Table S1. MS analysis Results of B1

Accession	Description
ORZ08349.1	hypothetical protein BCR42DRAFT_455530 [Absidia repens]
ORZ16986.1	phosphoesterase family-domain-containing protein [Absidia repens]
SAL96217.1	hypothetical protein [Absidia glauca]
SAM07131.1	hypothetical protein [Absidia glauca]
SAM08759.1	hypothetical protein [Absidia glauca]
ORZ06946.1	phosphoesterase family-domain-containing protein [Absidia repens]
SAM03680.1	hypothetical protein [Absidia glauca]
ORX55697.1	hypothetical protein DM01DRAFT_1335093 [Hesseltinella vesiculosa]

Table S2. MS analysis Results of B2

Accession	Description
ORZ07621.1	rhizopuspepsin 4 precursor [Absidia repens]
ORZ19634.1	phosphoesterase family-domain-containing protein [Absidia repens]
P81203.1	RecName: Full=Nuclease C1 - [NUC1_CUNEE]
SAM06107.1	hypothetical protein [Absidia glauca]
SAM00843.1	hypothetical protein [Absidia glauca]
SAM03620.1	hypothetical protein [Absidia glauca]
SAM00380.1	hypothetical protein [Absidia glauca]
ORZ09837.1	hypothetical protein BCR42DRAFT_423166 [Absidia repens]
CAA30805.1	actin, partial [Absidia glauca]
SAL99136.1	hypothetical protein [Absidia glauca]

Table S3. MS analysis Results of B3

Accession	Description

AMZ03504.1	actin, partial [Gongronella orasabula]
CAA30805.1	actin, partial [Absidia glauca]
ORZ23001.1	70 kDa heat shock protein 3 [Absidia repens]
ORZ19761.1	polyubiquitin, partial [Absidia repens]
SAM03706.1	hypothetical protein [Absidia glauca]
SAM04007.1	hypothetical protein [Absidia glauca]
ORZ07582.1	acyl transferase/acyl hydrolase/lysophospholipase [Absidia repens]
ORX61965.1	dynein heavy chain [Hesseltinella vesiculosa]
SAM09464.1	hypothetical protein [Absidia glauca]
ORX53586.1	chitin deacetylase [Hesseltinella vesiculosa]
P81203.1	RecName: Full=Nuclease C1 - [NUC1_CUNEE]
ORZ20708.1	thioredoxin-like protein [Absidia repens]
ORZ17063.1	hypothetical protein BCR42DRAFT_490992 [Absidia repens]
ORZ11203.1	Dolichyl-diphosphooligosaccharide--protein glycosyltransferase subunit WBP1 [Absidia repens]
ORZ09627.1	sorbitol dehydrogenase [Absidia repens]
ORX60347.1	cobW-domain-containing protein, partial [Hesseltinella vesiculosa]
ORX45707.1	hypothetical protein DM01DRAFT_1377999 [Hesseltinella vesiculosa]
SAL97209.1	hypothetical protein [Absidia glauca]

Table S4. Statistics of data output

Sample	CleanReads	CleanBase	CleanQ20	CleanQ30	CleanGC
U1	30371185	9078190036	96.61%	90.91%	37.22%
U2	29028881	8669454252	97.27%	92.31%	37.03%
U3	27552810	8226958809	97.48%	92.74%	37.33%
I1	52516835	15701338728	97.06%	91.80%	36.93%
I2	43527708	12975841178	97.44%	92.71%	38.13%
I3	47071335	14043692418	97.27%	92.34%	39.05%

Table S5. Statistics of differential gene number

DEG	Total	Down	Up
Induced _vs_ Uninduced	3051	1076	1975

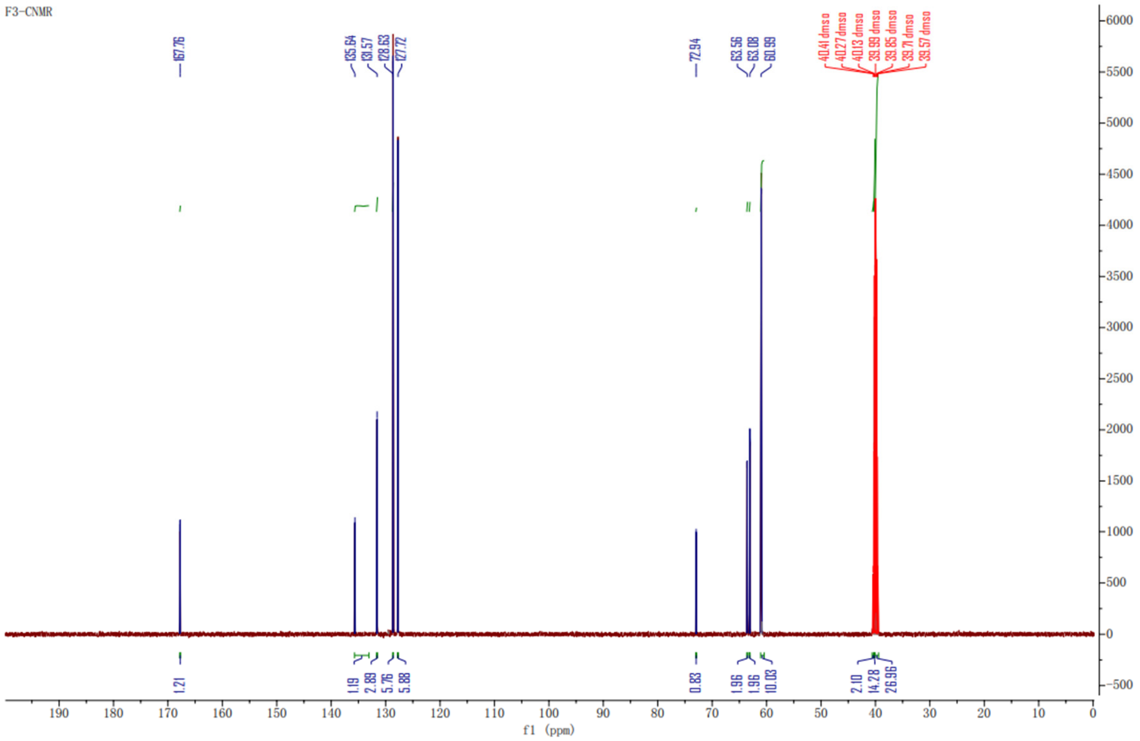

^{13}C NMR (151 MHz, dmso) δ 226.13, 167.76, 135.64, 131.57, 129.14, 128.63, 127.72, 72.94, 63.56, 63.08, 62.94, 60.99, 40.53, 40.41, 40.27, 40.13, 39.99, 39.85, 39.71, 39.57.

Figure S1. The ^{13}C NMR spectra of P2 (151 MHz, DMSO-*d6*).

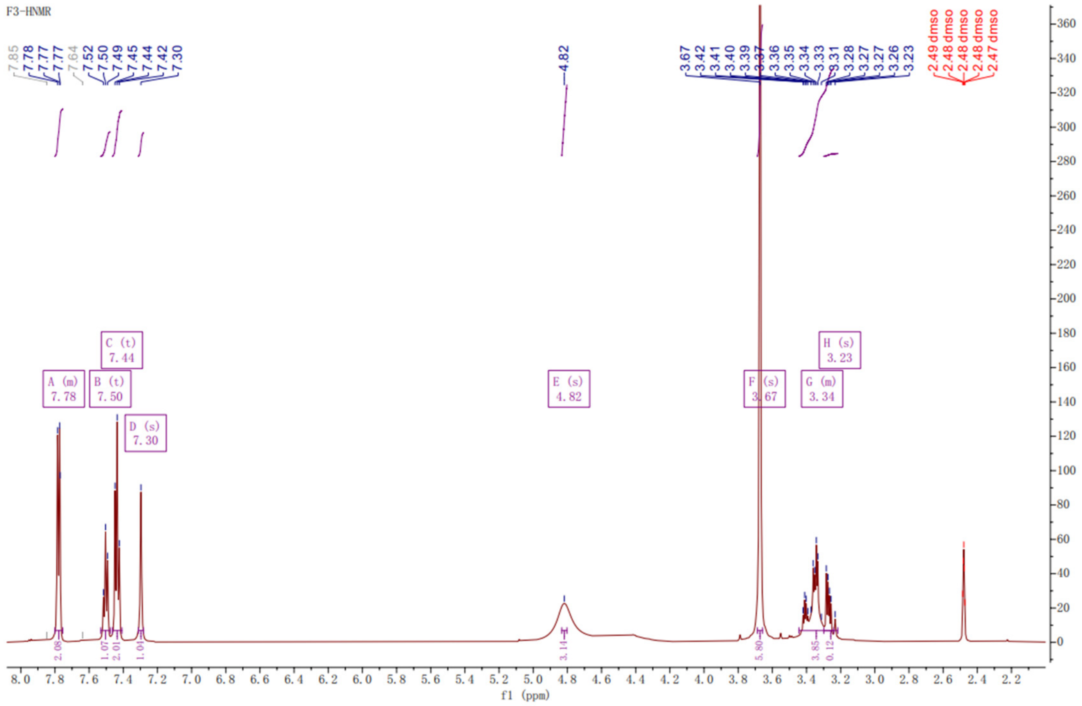

¹H NMR (600 MHz, dmso) δ 7.85, 7.78, 7.77, 7.77, 7.64, 7.52, 7.50, 7.49, 7.45, 7.44, 7.42, 7.30, 4.82, 3.67, 3.42, 3.41, 3.40, 3.39, 3.37, 3.36, 3.35, 3.34, 3.33, 3.31, 3.28, 3.27, 3.27, 3.26, 3.23, 2.49, 2.48, 2.48, 2.48, 2.47, 1.70, 1.21, 1.06.

Figure S2. The ¹H NMR spectra of P2 (600 MHz, DMSO-*d6*).

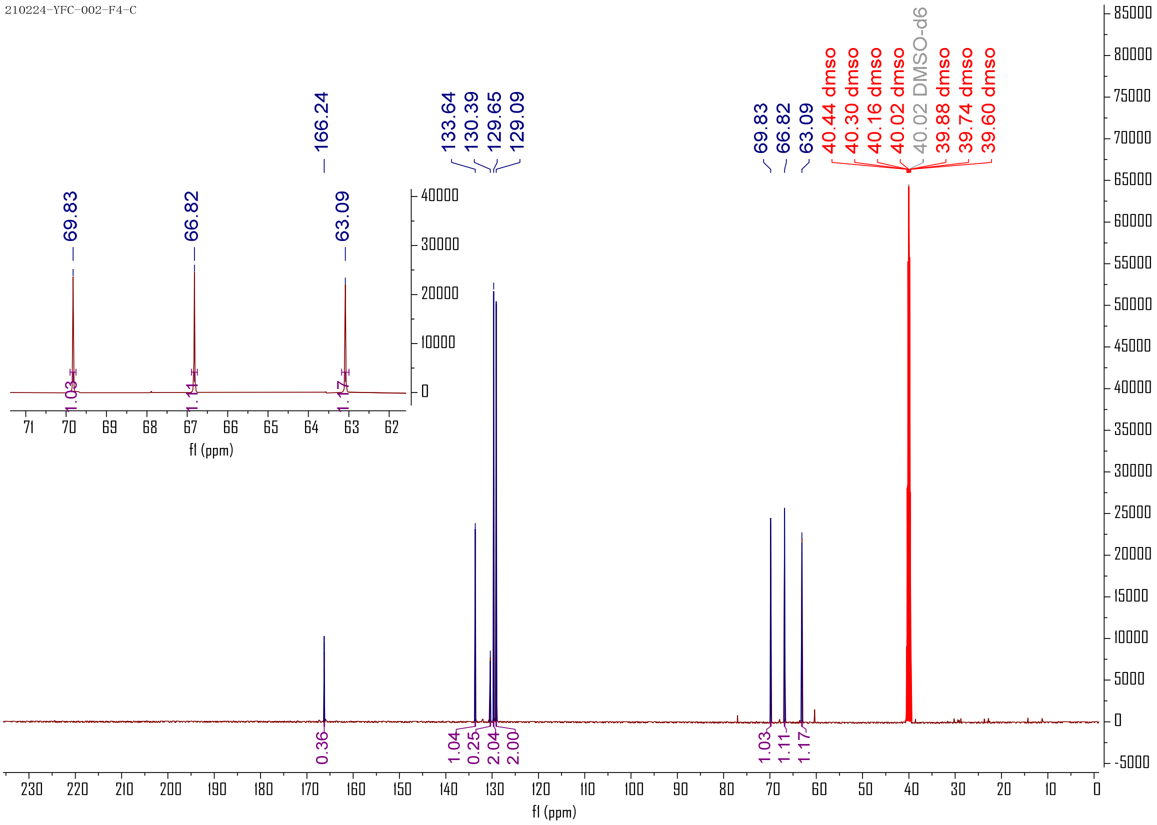

Figure S3. The ¹³C NMR spectra of P3 (151 MHz, DMSO-*d6*).

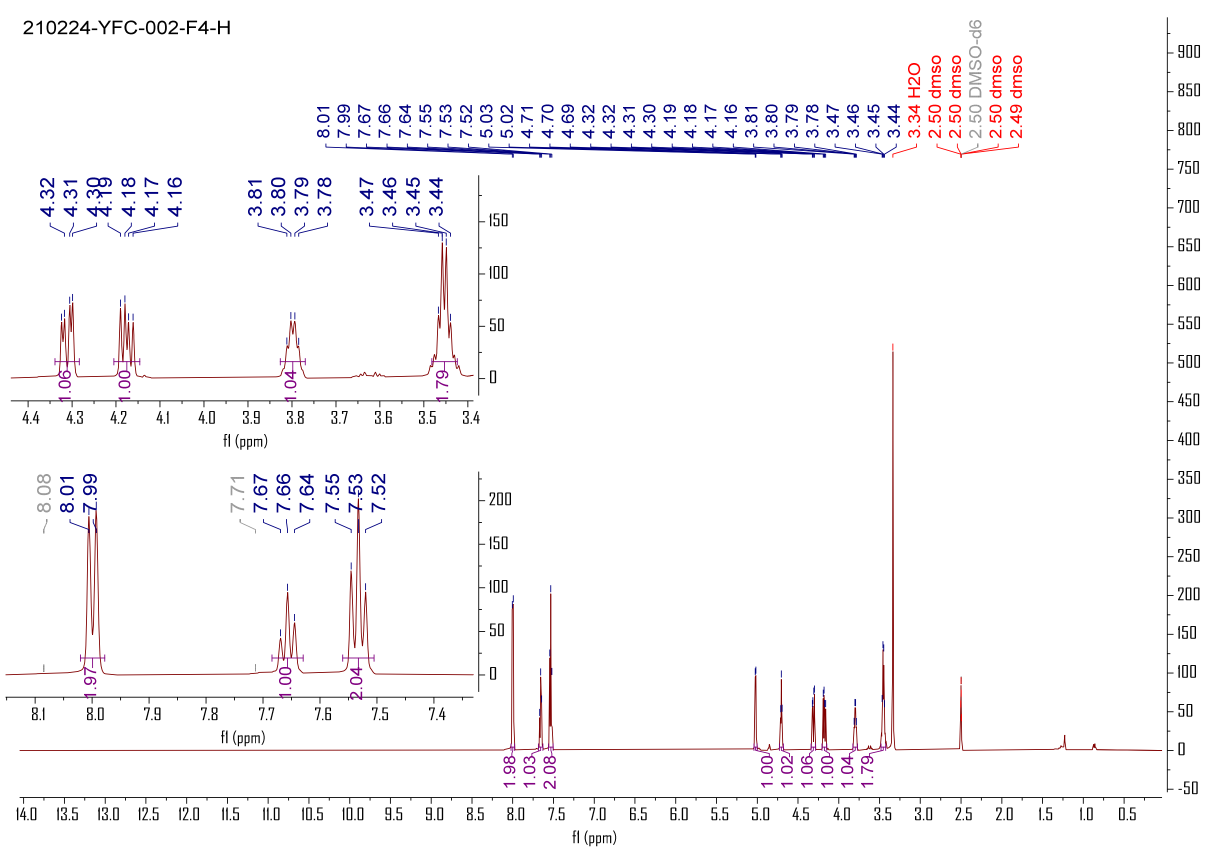

Figure S4. The ^{1}H NMR spectra of P2 (600 MHz, DMSO-*d6*).

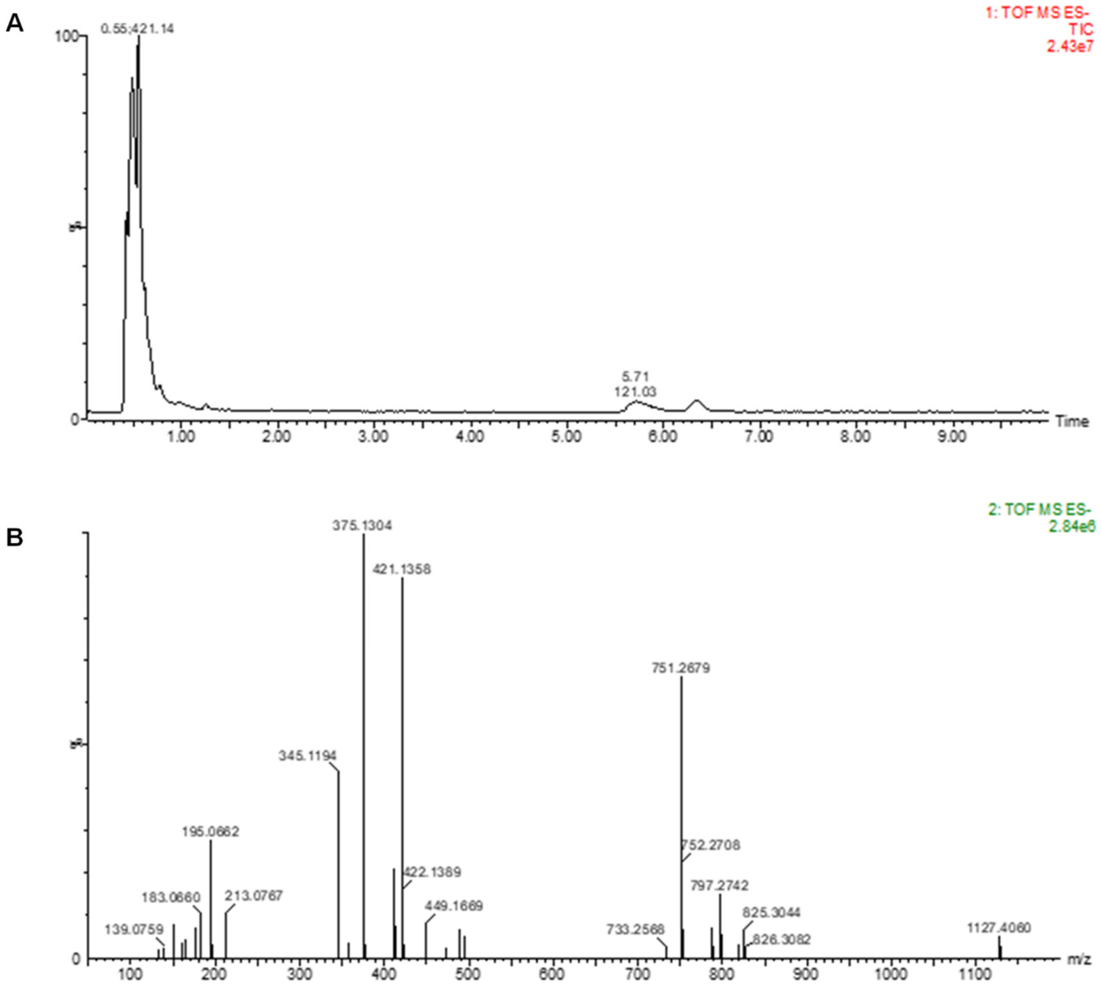

Figure S5. The mass spectra of component P1. A was the total ion flow diagram; B was the primary mass spectrum in negative ion modes.
